# Universal Biomaterial-on-Chip: a versatile platform for evaluating cellular responses on diverse biomaterial substrates

**DOI:** 10.1007/s10856-023-06771-x

**Published:** 2024-01-11

**Authors:** Abdul Raouf Atif, Morteza Aramesh, Sarah-Sophia Carter, Maria Tenje, Gemma Mestres

**Affiliations:** Division of Biomedical Engineering, Department of Materials Science and Engineering, Science for Life Laboratory, Uppsala University, 751 22 Uppsala, Sweden

## Abstract

Microfluidics has emerged as a promising approach for assessing cellular behavior in vitro, providing more physiologically relevant cell culture environments with dynamic flow and shear stresses. This study introduces the Universal Biomaterial-on-Chip (UBoC) device, which enables the evaluation of cell response on diverse biomaterial substrates in a 3D-printed microfluidic device. The UBoC platform offers mechanical stimulation of the cells and monitoring of their response on diverse biomaterials, enabling qualitative and quantitative in vitro analysis both on- and off-chip. Cell adhesion and proliferation were assessed to evaluate the biocompatibility of materials with different physical properties, while mechanical stimulation was performed to investigate shear-dependent calcium signaling in pre-osteoblasts. Moreover, the applicability of the UBoC platform in creating more complex in vitro models by culturing multiple cell types was demonstrated, establishing a dynamic multicellular environment to investigate cellular interfaces and their significance in biological processes. Overall, the UBoC presents an adaptable tool for in vitro evaluation of cellular behavior, offering opportunities for studying various biomaterials and cell interactions in microfluidic environments.

**Graphical Abstract:**

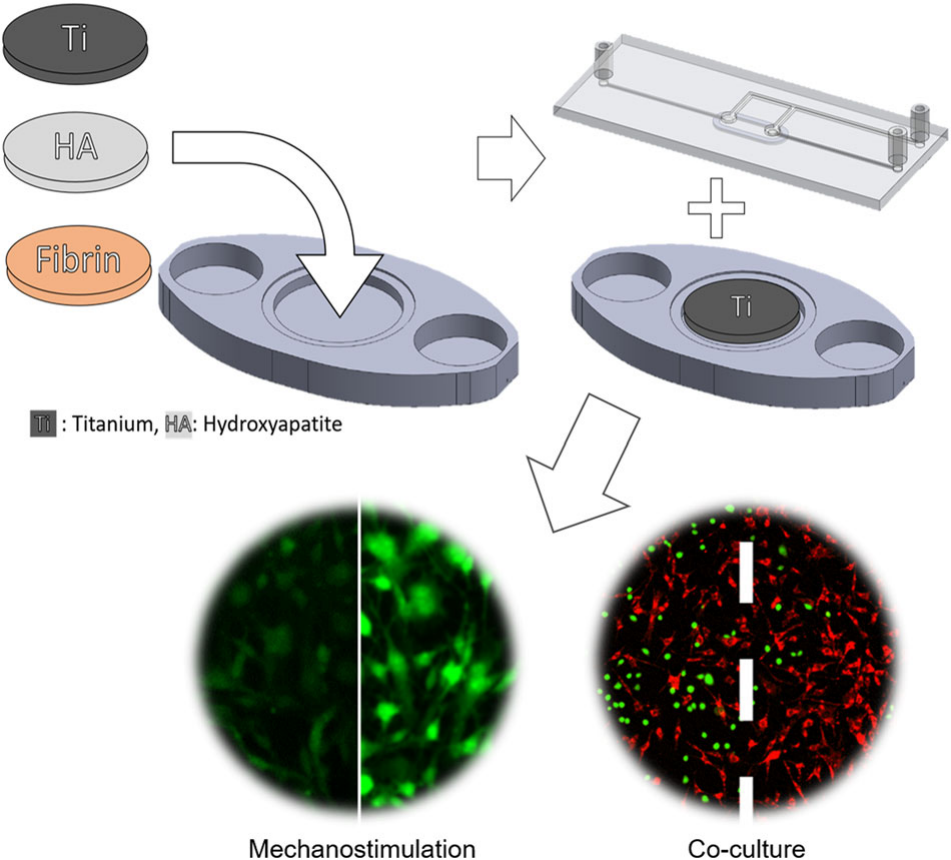

## Introduction

The development of biomaterials for biomedical applications relies on accurate and physiologically relevant in vitro testing systems to overcome the current discrepancies between in vivo and in vitro results [1]. One promising approach to address this challenge is the utilization of microfluidics, which enables the assessment of cellular behavior in more realistic cell culture environments by incorporating dynamic flow and shear stresses [2–5]. Microfluidic platforms offer advantages such as flexibility in device dimensions, constant media perfusion, and the ability to mimic physiological conditions [2–5].

In the context of biomaterials, microfluidics holds the potential to screen biomaterial libraries and evaluate their properties and cellular responses, including biocompatibility, proliferation, and cell migration [6–8]. However, existing microfluidic platforms are often designed for specific biomaterial types with similar properties and dimensions, resulting in limited comparability between platforms and difficulty in correlating results. Therefore, there is a need to develop a universal platform that can accommodate a wide range of biomaterials with varying properties under the same evaluation conditions.

To address this gap, this study introduces the Universal Biomaterial-on-Chip (UBoC) device, which enables the evaluation of cell culture performance on biomaterial substrates, namely calcium-deficient hydroxyapatite, titanium and fibrin. The UBoC device offers live cell stimulation and monitoring, allowing for qualitative and quantitative in vitro analysis both on- and off-chip. The functionality of the UBoC device was validated through cell culture experiments on different biomaterial substrates, assessment of cell adhesion and proliferation, and investigation of shear-dependent calcium signaling. In addition, the modularity of the UBoC platform was demonstrated by incorporation of co-culture experiments and enablement of the study of cellular interfaces in a multicellular environment.

## Results

**Fig. 1:**
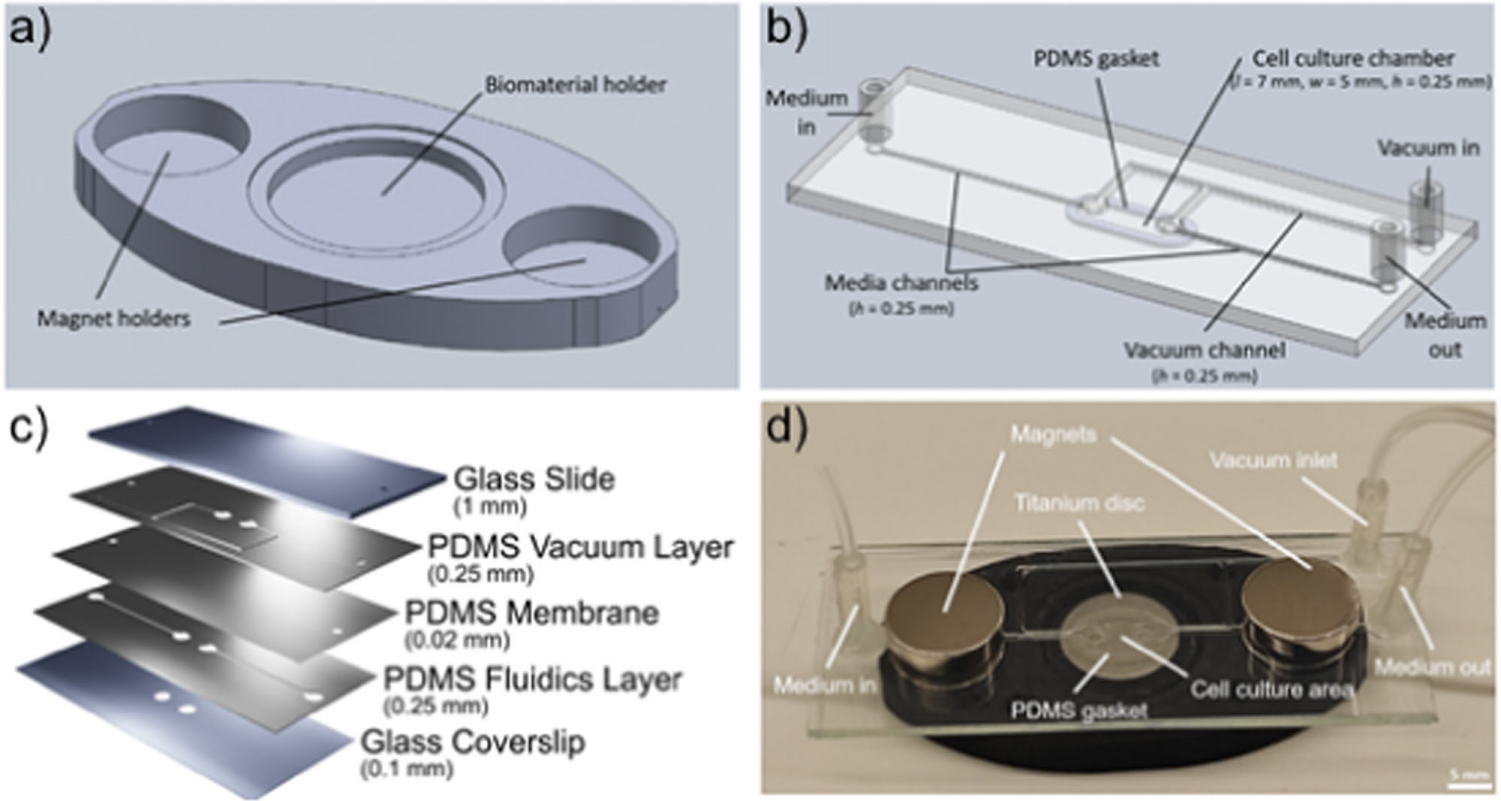
The Universal Biomaterial-on-Chip system. **a** Schematic of the substrate holder (lower layer) and **b** fluidics holder (upper layer). **c** Schematic of layer construction of the fluidics holder component. **d** Photograph of the UBoC device

The Universal Biomaterial-on-Chip (UBoC) device was developed for the examination of cell culture performance on biomaterials of distinctive properties and is illustrated in Fig. 1. The device is composed of two discrete components: Substrate holder (Fig. 1a) and the Fluidics holder (Fig. 1b). The substrate holder is fully 3D printed in a one-step process and is used to load the biomaterial of interest into the device. As for the fluidics holder, it is composed of multiple layers of plasma-bonded optically-clear PDMS and glass (Fig. 1c) and is used to deliver culture medium to the cells cultured on the loaded biomaterial. The substrate holder and fluidics holder are connected and sealed using a gasket-based sealing system, and are held together using neodymium magnets (Fig. 1d). The sealing process creates a channel over the biomaterial, enabling the buildup of a medium flow. Furthermore, a bubble trap and removal system are incorporated into the fluidics holder to prevent bubble formation and the corresponding induced cellular damage. While flow-based biomaterial in vitro culture systems were previously developed, the main advantage of the UBoC device is the extent of modularity and flexibility, offering versatility in selecting the biomaterial type and dimensions for incorporation [9, 10].

### Bubble trapping and removal for reliable cell culture

**Fig. 2:**
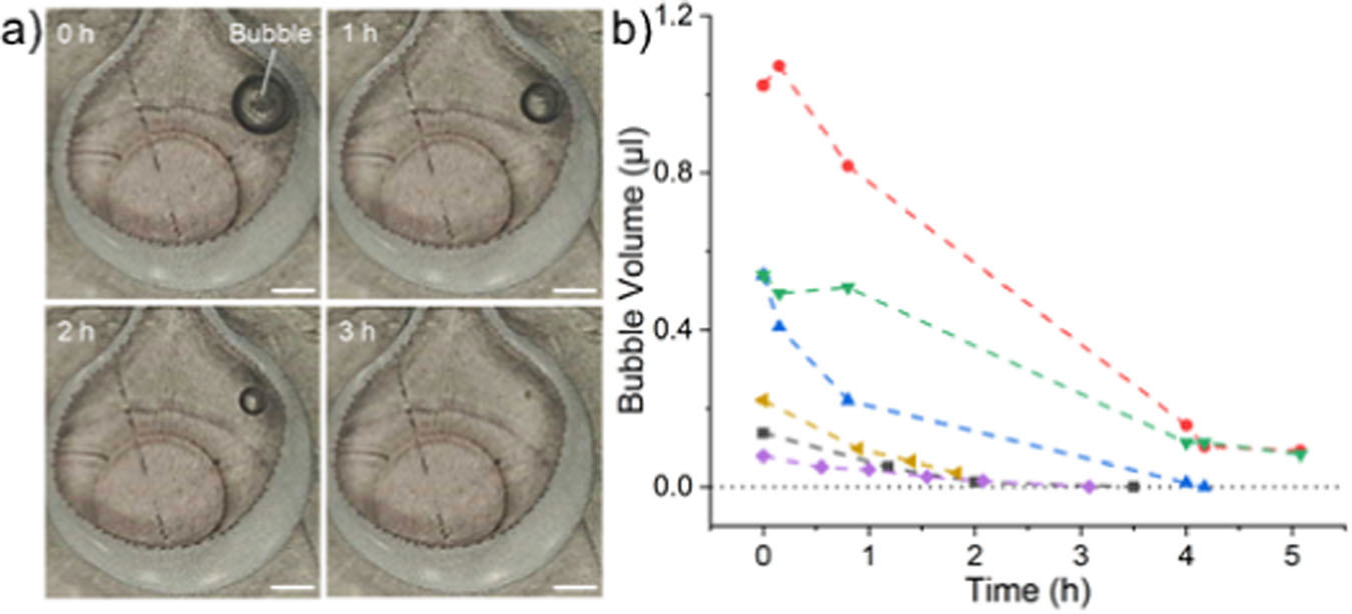
Bubble removal. **a** Time-lapse of representative bubble undergoing removal over a 3-h period. The bubble can be seen in the top-right of each photograph and can be observed to shrink in size as time passes. The scale bar is 0.5 mm. **b** Quantification of bubble removal rate, where each line corresponds to an individual air bubble that was tracked during the removal process

Within the UBoC device, an integrated in-line bubble removal system was added. By building up a vacuum environment against a 20 µm gas-permeable PDMS membrane, the system could remove trapped air at an average rate of 0.09 ± 0.05 µl h^−1^. While reliably successful at diminishing bubbles, there was variability in the removal rate, with larger bubbles experiencing faster removal rates compared to smaller bubbles (Fig. 2). By placing the bubble trap just before the culture area, we surmise that all potential sources of bubbles were accounted for (e.g., medium reservoir and preceding tubing interconnections). Another advantage is that unlike other approaches, flow in the UBoC device was uninterrupted throughout the bubble removal process, thereby not affecting the ongoing cell culture [11]. Despite the slower removal rate in the UBoC device compared to other debubbler designs, the design presented in this work performed aptly at its intended function [12, 13].

### Integration of different biomaterials in UBoC for cell culture applications

**Fig. 3:**
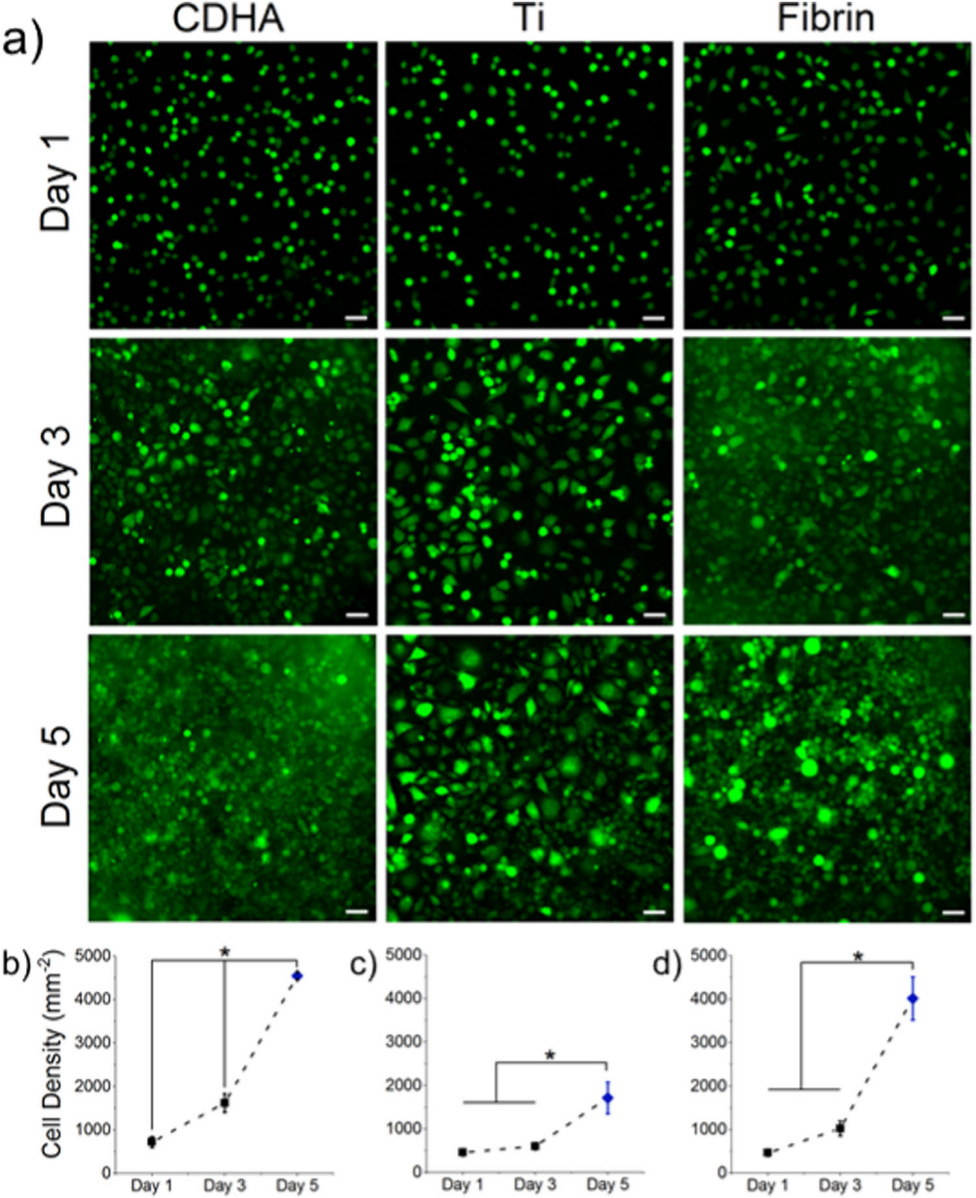
Cell culture on different biomaterials in UBoC. **a** L929 Fibroblast cell culture on CDHA, Ti and fibrin substrates. Images were taken at day 1, 3 and 5 of culture. Cells were stained with cell tracker green for live monitoring. Scale bar corresponds to 100 µm. **b** Quantification of cell counts presented as cell density (cells mm^−2^) for CDHA, **c** Ti and **d** fibrin over 5 days. Interval bars indicate standard deviation and * indicates significance between compared samples (*p* < 0.05). Day 5 samples indicated as blue diamond symbols due to counting of nuclei rather than cytoplasm

The UBoC device is compliant to biomaterials of different characteristics and dimensions. To demonstrate this, cell culture studies were performed on three different types of materials. Fibroblast cells (L929) were cultured on integrated calcium-deficient hydroxyapatite (CDHA), titanium (Ti) and fibrin gel substrates for a period of 5 days under a continuous 2 µl min^−1^ flow. The available area for culture was 0.32 cm^2^, which matches the culture area of a conventional 96-well plate. As observed on day 1 of culture, fibroblasts successfully adhered on all tested substrates (Fig. 3a). Furthermore, cells proliferated on the tested substrates over the course of the experiment with similar growth rates observed for CDHA and fibrin, while cells on Ti proliferated at a lower rate. Notably, cell counts on day 5 were obtained via nuclei rather than cytoplasmic counts (as was done on days 1 and 3) due to cell confluency, so a certain degree of overestimation is expected. Nevertheless, cell counts obtained on day 5 were validated via a quantitative LDH activity assay, which confirmed the trend observed from direct cell counting (Supplementary Fig. S1).

### Live cell stimulation and monitoring—flow-dependent Ca^2+^ signaling in pre-osteoblasts

**Fig. 4:**
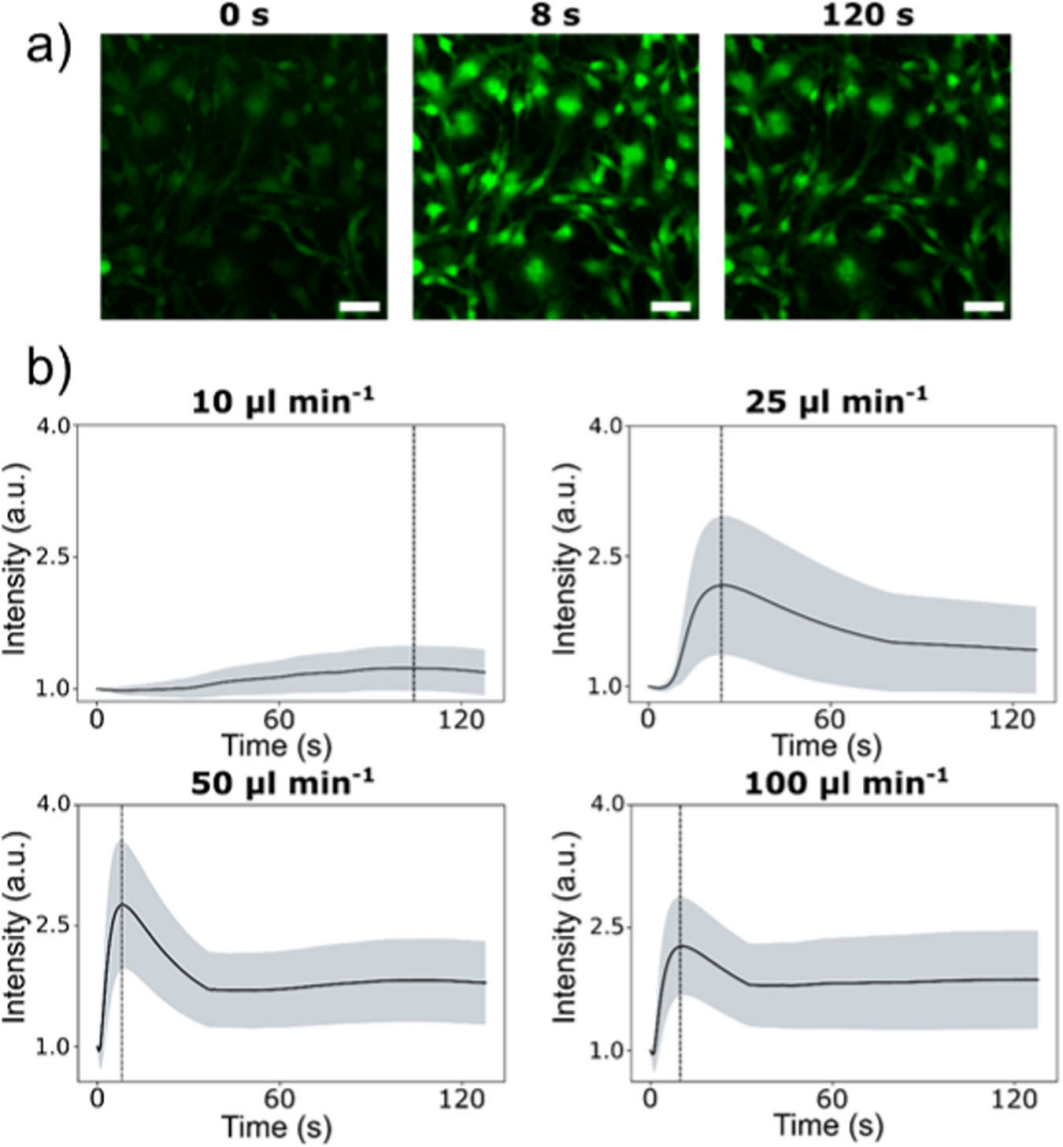
Quantification of the effect of applied fluid flow on intracellular Ca^2+^ response in pre-osteoblast cells. **a** Representative images of labeled cells undergoing a continuous 50 µl min^−1^ stimulation at time points of 0, 8 (peak intensity) and 120 s. Scale bar corresponds to 50 µm. **b** Normalized averaged signal intensities collected over the experimental duration for all 4 samples. The line indicates averaged signal and the shaded area indicates the standard deviation at a given time point. The dotted line indicates the peak intensity for a given flow condition

Using the UBoC device, the intracellular Ca^2+^ flux of pre-osteoblasts under varying flow conditions was investigated. A range of flow rates from 10 to 100 µl min^−1^ (approximately corresponding to shear stresses of 0.03–0.33 dyn cm^−2^) were applied for 2 min. As shown in Fig. 4a, there was an increase in Ca^2+^ release after the onset of the flow stimulation, measured via fluorescence intensity of a Ca^2+^-sensitive dye (Calbryte™ 520 AM). It can generally be noted that greater fluorescence peak intensities resulted in increased flow rates (Fig. 4b). For flow stimulations of 25 µl min^−1^ and above, there was an initial spike in fluorescence intensity approximately within 22 s, followed by a stable elevated fluorescence intensity level. The strongest response was observed for the 50 µl min^−1^ flow-stimulated samples, which reached a maximal average intensity of 2.77 ± 0.81 folds. Interestingly, these flow conditions generated a stronger response compared to the 100 µl min^−1^ flow, which reached peak intensities of 2.40 ± 0.56, thereby potentially indicating stimulation saturation at flow rates above 50 µl min^−1^.

### Fibroblasts and pre-osteoblasts interface co-culturing under laminar flow

**Fig. 5:**
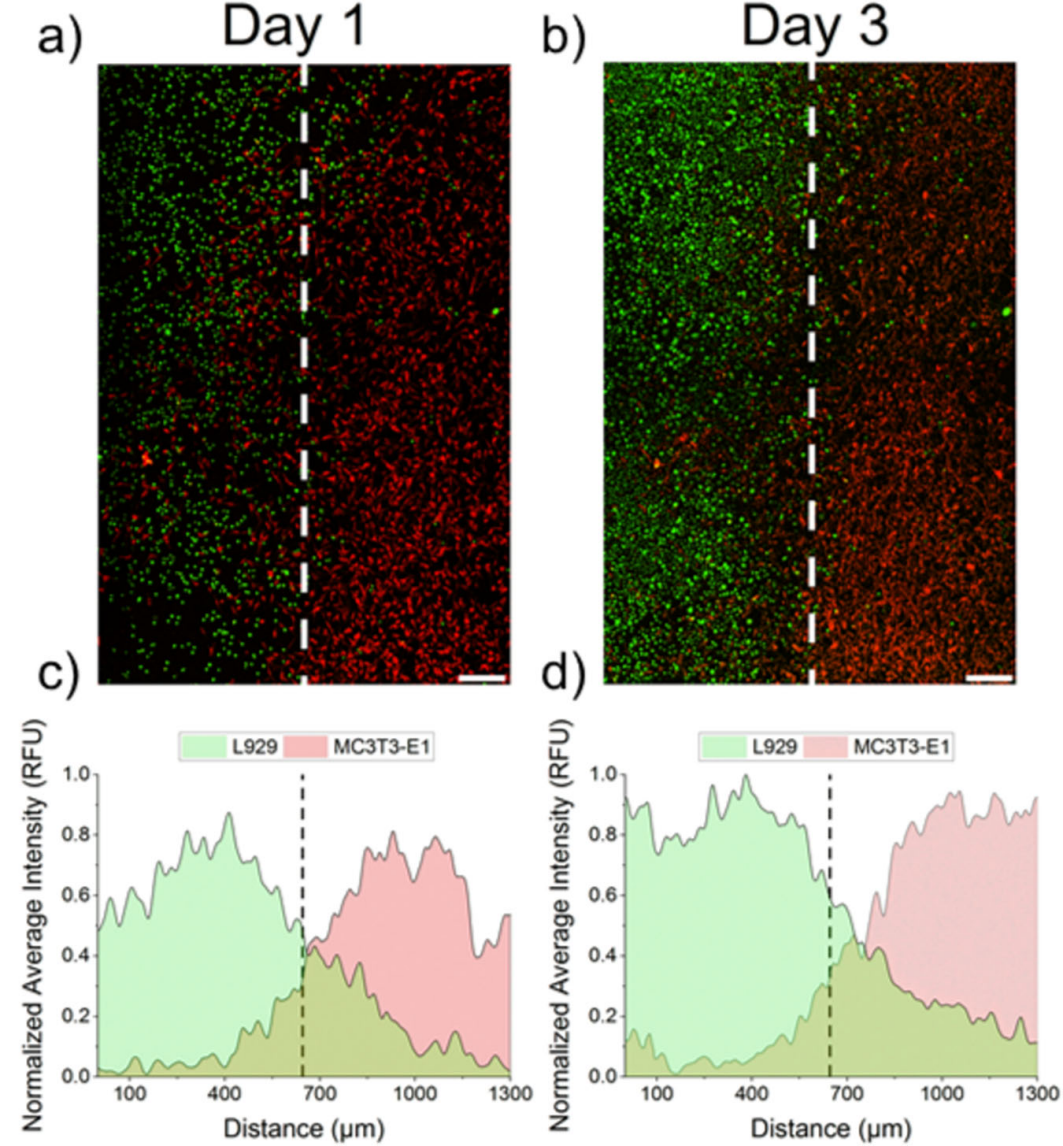
Co-culturing cells on UBOC. **a** Fluorescence microscope images of the co-culture of L929 fibroblasts (green) and MC3T3-E1 pre-osteoblasts (red) on a Ti substrate integrated in a UBoC device on day 1 and **b** day 3 of co-culture (of the same sample). Scale bar corresponds to 500 µm. **c** Quantitative depictions of the average cellular distribution of both cell types were plotted for both day 1 and **d** day 3 of culture. The dashed line indicates the central vertical axis of the chip

The modular design of the UBoC device enables further functionality via simple design adjustments. Employing a customized fluidics holder (Supplementary Fig. S3), we successfully co-seeded fibroblasts and pre-osteoblast cells with uniform cell densities (20,000 cells cm^−2^) under laminar flow conditions. This design allowed parallel flow streams within the chip with minimal mixing, while also enabling distinct culture media delivery to each cell type (i.e., α-MEM to pre-osteoblasts and MEM to fibroblasts). As demonstrated in Fig. 5a, both cell populations were effectively seeded within their designated flow streams. Even as both cell types underwent proliferation, the spatial distribution remained consistent up to day 3 of culture (Fig. 5b). Notably, a gradient in cellular distribution was evident, with maximal cell density observed at the edges of the culture area and gradually decreasing toward the center (Fig. 5c, d).

## Discussion

The UBoC device was developed as a tool for simplified in vitro investigations of different parameters (such as biocompatibility) of biomaterial substrates. Specifically, the use of UBoC places a reversible microfluidic channel directly onto the surface of the material, where cells can then be seeded or analytical material be collected. UBoC is compatible with a wide range of substrates of varying properties, wherein this work compatibility with CDHA, titanium and fibrin materials was demonstrated.

Specifically, within CDHA-integrated UBoC devices, the proliferation of fibroblasts exhibited significant enhancement under flow conditions (Fig. 3b), illustrating the potential mitigation of ionic exchange effects in the tested material. CDHA is a biomimetic bone cement that has been shown to induce changes in ionic composition of the surrounding medium, which has been associated with challenges to cell viability [10, 14–18]. However, the dynamic flow characteristics of the UBoC, superficially resembling in vivo circulations, could counteract ion composition effects, thereby enhancing cell growth and viability. Furthermore, Ti displayed an increase in fibroblast cell count during the 5-day culture period (Fig. 3c), indicating its biocompatibility. This aligns with the previous results, wherein pre-osteoblasts cultured on Ti within a microfluidic device exhibited notable proliferation over a 10-day culture period [9]. Lastly, fibroblast cells on fibrin showed strong proliferation in the UBoC device (Fig. 3d). These results were in line with work by Hokugo et al., who presented similar strong proliferative behavior for fibroblasts on a fibrin scaffold, with a ten-fold increase in total cell number over a 7-day period [19]. Notably, incorporating fibrin into microfluidic chips poses challenges due to its susceptibility to evaporation and detachment. Using the current approach, gel cross-linking was conducted off-chip within a cartridge module before insertion into the UBoC, facilitating integrability (Supplementary Fig. S2). All together, these results confirm the integrability of CDHA, Ti and fibrin in UBoC devices and their biocompatibility in the UBoC device culture environment.

In addition to sustaining cell culture, the UBoC device opens up the possibility of live cell stimulation and optical monitoring. To exemplify this, the sensitivity of pre-osteoblasts (stained with a calcium indicator dye) to shear stimulation was investigated. Pre-osteoblasts were used due to their association with the lacunar/canicular network (LCN), a major component of bone tissue matrix composed of small channels that generate flow during loading [20, 21]. When seeded on Ti within the UBoC, the cells displayed fluorescence that increased in intensity up to a 50 µl min^−1^ stimulation, after which the response appeared to plateau. The generated response is assumed to be linked to changes in intracellular calcium concentrations in osteoblasts, which is expected as calcium (Ca^2+^) is an important secondary messenger that is involved in multiple bone tissue processes, such as mechanotransduction, differentiation and mineralization [22–27]. Flow-dependent Ca^2+^ signaling in pre-osteoblasts may originate from different mechanosensitive mechanisms, such as from endoplasmic reticulum (ER) intracellular stores, actin cytoskeleton rearrangements or Piezo1 plasma membrane proteins [28, 29]. A combination of these factors may contribute to the flow-dependent Ca^2+^ signaling responses observed in this study and need to be investigated for further insight into the detailed mechanism.

Furthermore, the UBoC presents multiple advantages in comparison to alternative approaches for microfluidic interface co-culturing, such as surface patterning approaches or using surfaces with differing degrees of stiffness [30, 31]. As demonstrated with pre-osteoblast and fibroblast cell lines, interface co-culturing with UBoC is solely based on the principle of laminar flow and does not rely on surface modifications such as protein coatings or chemical treatment. This characteristic thereby makes UBoC comparatively more accessible, reliable and scalable method. For instance, the potential addition of more inlets and outlets would allow for the introduction of a wider range of cell types. As such, the UBoC device enables co-culturing of different cell types, opening the possibility of a concurrent investigation of cell–cell and cell–substrate interactions within a precisely controlled shear stress environment.

## Conclusion

With the growing use of microfluidic devices for biological applications, there is an emerging demand for standardization and modularity in design, particularly in the context of biomaterial research to enable impartial comparisons across diverse studies. The Universal Biomaterial-on-Chip (UBoC) device, presented in this study, introduces a versatile platform for flow-based studies involving cells cultured on diverse biomaterial substrates of varying sizes, stiffnesses, or shapes. The UBoC’s culture area can be designed to be aligned with standard 96-well culture plates. Furthermore, the UBoC’s capabilities for real-time live cell monitoring were validated by probing the mechanosensitivity of pre-osteoblasts through Ca^2+^ signaling, revealing a shear-dependent response within a range of 10–100 µl min^−1^, where the peak response was observed under a flow rate of 50 µl min^−1^, corresponding to a shear of approximately 0.17 dyn cm^−2^. Lastly, the UBoC device was slightly adapted to support interface co-culturing, where a co-culture of fibroblasts and pre-osteoblasts was demonstrated, both exhibiting proliferative behavior under the flow supply of their distinct culture medium. In summary, the UBoC platform stands as a promising tool with significant applicability in studying flow-induced mechanotransduction on biomaterials, biomaterial screening, and investigations into cell–cell and cell–biomaterial interactions. Its capacity for both quantitative and qualitative analyses on-chip and off-chip enables the evaluation of a wide array of in vitro assays on diverse biomaterials.

## Methods

### UBoC fabrication and material preparation

#### Fabrication of the UBoC device

The Universal Biomaterial-on-Chip (UBoC, Fig. 1) is a modular microfluidic device that was designed and built to enable in vitro cell culture on different biomaterials of varying dimensions and physical properties. The device is composed of two main components: The substrate holder (holds the integrated biomaterial in place) and fluidics holder (contains channels for fluid flow). The substrate holder houses the biomaterial as well as two magnets which serve as the locking mechanism for the device (Fig. 1a). An additional, smaller holder (named cartridge) that contains a cavity (*l* = 6.3 mm, *w* = 4.3 mm, *h* = 1 mm) and fits into the substrate holder was also prepared, specifically for use with soft (e.g., fibrin) and porous materials (e.g., hydroxyapatite) (Supplementary Fig. S2). The substrate holder and cartridge were constructed via additive manufacturing of PLA (Polylactic acid, 2.85 mm filament, #1613, 3DVerkstan) using a Fused Filament Fabrication (FFF)-based 3D printer (Ultimaker 2+, Ultimaker). The main settings used for the print were 0.1 mm layer height with 100% zigzag-patterned infill density. The models used for the print are shown in Fig. 1a and Supplementary Fig. S2 and can be found as STL file attachments to this publication. Depending on the dimensions of the biomaterial under study, the biomaterial holder area in the substrate holder can be adjusted prior to the print (in terms of height, width and shape) to best fit the incoming biomaterial sample. Subsequent to the print, neodymium magnets (⌀ = 12 mm, *h* = 3 mm, Magnordic) were glued (Instant adhesive 420, Loctite) into their respective slots in the opposing ends of the substrate holder.

The fluidics holder consists of channels that deliver growth medium to the cell culture area present on the biomaterial (Fig. 1b) and also contains an embedded cavity that enables a vacuum to be built up above the fluidics channels. This cavity is separated from the fluidic channels by a thin gas-permeable Polydimethylsiloxane (PDMS) membrane (*h* = 20 µm, Elastosil® Film 2030, Silex Silicones) and functions as a bubble trap to remove of trapped air from the fluid.

In order to fabricate the fluidics holder, PDMS sheets (*h* = 250 µm, Silex Silicones) were cut using a vinyl cutter plotter (CAMM-1 GS-24, Roland) to generate patterned PDMS pieces consisting of the fluidics layer and the vacuum layer. The gas-permeable membrane was fabricated in the same manner, but with a 20 µm PDMS sheet used instead. The cut PDMS layers were then surface treated using plasma under low pressure for 1 min (100% intensity, Atto Plasma Cleaner, Diener electronic GmbH) and then carefully aligned and bonded to each other in the following order: vacuum layer to membrane layer to fluidics layer (Fig. 1c). A glass slide (*h* = 1 mm, #631-1550, VWR) with pre-drilled holes (corresponding to inlets and outlets of the fluidics channels and gas cavity) was plasma-treated for 1 min and then bonded to the PDMS vacuum layer. To the inlet and outlet holes, silicone tubing (I.D. = 1 mm, O.D. = 3 mm, *l* = 10 mm, #228-0701P, VWR) was glued to serve as plug-and-play connectors to external tubing connections (e.g., media source, vacuum source). On the opposing side of the chip (i.e., fluidics layer side), a glass coverslip (24 × 60 mm^2^, #2, #630-2895, VWR) was plasma-treated for 1 min and then bonded to the PDMS surface. Prior to bonding, two 0.75 mm holes were etched in the glass coverslip (connecting fluidic channels and the culture area) using a 5 W, 532 nm laser operated at 70% power, 10,000 Hz Shot Frequency and 300 passes (AIO, ÖSTLING Märksystem AB). Finally, an oval-shaped PDMS gasket (*l* = 8 mm, *w* = 4.5 mm, *h* = 0.25 mm) was placed on the glass coverslip around the two etched holes, thereby defining the cell culture area. The gasket was held in place via frictional forces against the glass. To better illustrate the fabrication of the fluidics holder, a schematic breakdown of the layers is shown in Fig. 1c. A photograph of the complete device is shown in Fig. 1d.

#### Preparation of biomaterials

Calcium-deficient hydroxyapatite (CDHA) was prepared from a cementitious reaction using α-tricalcium phosphate (α-TCP, Ca_3_(PO_4_)_2_) as the starting solid phase substrate. α-TCP was produced via mixing calcium carbonate (CaCO_3_, #10687192, Acros Organics) and dicalcium hydrogen phosphate anhydrous (CaHPO_4_, #40232.30, Alfa Aesar) in a 1:2 molar ratio. The mixture was then heated for 5 h in a furnace (MF 4/16, Entech) at 1450 °C on a zirconia plate setter (with a total heat treatment time, including ramp-up and ramp-down, of 18 h) and then quenched in air. The resulting powder was then dry milled at 300 rpm for 15 min using a planetary ball mill (PM 400, Retsch). Per 100 g of powder, 100 zirconia-milling balls (⌀ = 10 mm) were used.

The α-TCP powder (solid phase) was then mixed with a 2.5% disodium hydrogen phosphate (Na_2_HPO_4_) in Milli-Q water (liquid phase) at a liquid-to-powder ratio of 0.65 ml g^−1^. Thereafter, the mix was poured into the oval-shaped cavity within the 3D-printed cartridge module and set for up to 4 h in an incubator at 37 °C and 100% relative humidity. After the setting process, the pieces were transferred into a 0.9% sodium chloride (NaCl) solution at 37 °C for 10 days to allow for complete conversion into CDHA. The purity of the α-TCP powder and resultant CDHA was confirmed by X-ray diffraction [10].

Medical-grade (Grade 5) titanium alloy (Ti_6_Al_4_V) discs (⌀ = 12 mm, *h* = 1 mm) were purchased (Optimel Plåtteknik AB). The Titanium alloy will be referred to as “Ti” within the publication.

Fibrin was also used as a biomaterial model for cell culture. To prepare fibrin, equal volumes of fibrinogen (10 µg ml^−1^, #341573, Merck) and thrombin (1 U ml^−1^, #T7326, Merck) in phosphate-buffered saline (PBS, #14200067, Fischer Scientific) were pipetted into a 3D-printed cartridge module cavity (*l* = 6.3 mm, *w* = 4.3 mm, *h* = 0.3 mm). The two protein solutions were briefly mixed and then allowed to polymerize and gelate for 3 min within a laminar flow hood at room temperature and humidity, after which culture medium was added above the gel to prevent drying.

#### Bubble trap characterization

In order to verify the functionality of the in-line bubble trap within the UBoC device, its ability to remove bubbles was characterized. To do so, a portable digital USB microscope (AM3113T, Dino-Lite) was mounted inside of a cell culture incubator. Using TYGON® tubing (I.D. = 0.51 mm, O.D. = 1.52 mm, ND 100-80, Saint-Gobain), a UBoC device was connected to a reservoir (T25 flask containing culture medium) and a glass syringe (1 ml) connected to a syringe pump (Nemesys, Cetoni) via the chip’s inlet and outlet, respectively. In addition, the vacuum inlet of the UBoC device was connected to a separate syringe (1 ml) attached to the same syringe pump. After connecting all the necessary tubing, the UBoC device was placed within the microscope’s field of view and focused on the bubble trap area of the chip. The syringe pumps were then started with the medium flow set at a continuous rate of 2 µl min^−1^ and the vacuum flow set at a rate of 10 µl min^−1^. The vacuum flow was run for 10 min at 30-min intervals. By temporarily detaching and reattaching the chip’s inlet medium supply tubing, a bubble was introduced into the chip, which was promptly captured by the bubble trap in the chip. Images of the trapped bubble were then taken by the USB microscope at 1 h intervals. Six separate bubble replicates were imaged. The diameter size of the bubble was quantified via Fiji ImageJ and used to calculate the volume of the bubble at a given time [32].

#### Sterilization and pre-experimental preparation

Prior to cell culture, the UBoC device components and associated tubing were sterilized to prevent any bacterial or fungal contamination. For CDHA and Ti, a 70% ethanol solution was flowed through the UBoC device with the integrated biomaterial and allowed to treat the channels for up to 2 h. As for fibrin, the cartridge module was initially loaded as empty into the UBoC device and sterilized. Within a sterile laminar flow hood, the fibrin gel was later prepared and added to the cartridge after sterilization via opening the UBoC device temporarily.

For CDHA and Ti, the ethanol solution was washed away by flushing the UBoC device with autoclaved PBS solution (100 µl). The device was then flushed with cell culture medium (100 µl) corresponding to the cell line to be seeded. Notably for CDHA-loaded UBoC devices, the corresponding culture medium was flowed in at rate of 1 µl min^−1^ for up to 24 h prior to seeding to allow protein coating of the CDHA surface to ensure optimal cell attachment [16].

### Cell culture

L929 mouse fibroblasts (ECACC) and MC3T3-E1 mouse pre-osteoblasts (ATTC, subclone 14) are commonly used for cytotoxicity testing [33, 34]. L929 cells were maintained in MEM (#11965126, Thermo Fischer Scientific) supplemented with 10% Fetal Bovine Serum (FBS Hyclone, #SV3016003, GE Healthcare) and 1% Penicillin/Streptomycin (P/S, #DE17-602E, BioWhittaker). MC3T3-E1 cells were maintained in MEM-α medium (Gibco, #A1049001, Thermo Fischer Scientific), which was supplemented with 10% FBS and 1% P/S. During experimental work, Hyclone MEM-α (#SH3026501) with 10% FBS and 1% P/S supplement was used as MC3T3-E1 culture medium. Gibco MEM-α was used as a maintenance medium as it is ascorbic acid-free, which would otherwise bias cells toward a differentiation phenotype [35]. Both cell types were split once a confluence level of approximately 70% was reached, with the split performed using TrypLE^TM^ (#12605028, Thermo Fisher Scientific) for 3 min. Both cell types were used between a passage of 15 and 25 (cells were received at passage 15). Cell culture was performed in an incubator (NB-202XL, N-Biotek) at 37 °C and a 5% CO_2_ environment.

### Characterization of UBoC capabilities

#### Cell culture on different materials integrated in UBoC device

Prior to seeding, L929 fibroblasts were stained in suspension with a green CMFDA dye (15 µM, C2925, Invitrogen) solution in clear MEM (#51200046, Thermo Fischer Scientific) for 30 min. Using a pipette, cell seeding solution (45 µl) containing 20,000 cells cm^−2^ was flowed into each UBoC device. Subsequent to seeding, the UBoC devices were transferred to an incubator and the inlet and outlet of UBoC devices were connected to a flow set-up consisting of a medium reservoir and a syringe pump. On all substrates, cells were allowed to adhere for 1 h to ensure attachment to the biomaterial surface. A flow of 2 µl min^−1^ was then started and run over a period of 5 days. To account for trapped bubbles, the vacuum inlet of the UBoC device was also connected to a syringe that operated at a pull flow of 10 µl min^−1^ for 10 min every 30 min. Triplicate UBoC devices were prepared for each biomaterial sample, as well as a well plate positive control prepared with the same seeding density of 20,000 cells cm^−2^ and culture medium (200 µl) that was refreshed daily.

Seeded UBoC devices were imaged on days 1, 3 and 5 of culture. To reduce the chance of bubble formation or infection during imaging, the inlets and outlets of the UBoC device were blocked with stoppers. At least five representative images were taken of each device by methodically imaging the edge and center sections of the UBoC culture area using a fluorescence microscope (IX73 inverted microscope, Olympus). On day 5 of culture, cells growing in the UBoC device were further stained with calcein (1 µg ml^−1^, #C3099, Thermo Fisher Scientific), propidium iodide (0.7 µg ml^−1^, #P3566, Thermo Fisher Scientific) and Hoechst 33342 (0.5 µg ml^−1^, #11534886, Thermo Fischer Scientific) to stain the live cells (cytoplasm), dead cells (nuclei) and nuclei, respectively. The captured images corresponding to each dye were merged to generate a representative image with the individual stains overlaid. Using Fiji ImageJ, the cells were manually counted and averaged for each condition, with resultant numbers conveying the degree of proliferation for each cell type on each tested biomaterial [32]. The cell images taken on days 1 and 3 were counted using the cytoplasmic stain as reference, but due to confluence, cell images on day 5 were counted using the nuclear stain as reference. To reduce background noise, the images were treated with a rolling ball filter (radius = 20 pixels). Triplicates were taken for each sample and the entire experiment was performed twice.

Following imaging on day 5, an indirect method consisting of cell lysis and subsequent assay for lactate dehydrogenase (LDH), was performed to confirm cell count data. This was carried out using an analysis kit following the manufacturer’s instructions (TOX7 Kit, Sigma-Aldrich). Briefly, the cells were washed by flushing PBS (100 µl) through the UBoC device. The PBS was then replaced with a 10% v/v lysis buffer solution (40 µl, TOX7 Kit, Sigma-Aldrich) and left in an incubator at 37 °C for 50 min. The resultant lysate solution (40 µl) was collected and diluted to a final volume of 100 µl. The samples were further diluted by ten-fold in 10% v/v lysis buffer solution to avoid signal saturation. Each sample (50 µl) was then aliquoted into a 96-well plate. To these wells, LDH assay reaction mix (100 µl, prepared via mixing equal volumes of substrate, dye and cofactor solutions) was added. The reaction was then allowed to proceed in the dark for 20 min at room temperature. Absorbance values at 490 nm (signal) and 690 nm (background) were then read for each sample using a microplate reader (Spark, Tecan). For each sample, triplicates were taken and the result was averaged. The entire experiment was done twice.

#### Capture of flow-response behavior of MC3T3-E1 via Ca^2+^-sensitive dye

MC3T3-E1 pre-osteoblasts were seeded on Ti-integrated UBoC devices at a density of 20,000 cells cm^−2^. Ti was used as the model biomaterial due to its relatively smooth surface and homogeneity between samples. Subsequently, the devices were transferred to an incubator and connected via the inlet and outlet to the flow set-up and vacuum set-up described previously (section 4.3.1). The cells were allowed to adhere for 1 h before a medium flow of 2 µl min^−1^ was started. The vacuum flow used was set at 10 µl min^−1^ and run for 10 min every 30 min.

After 24 h of culture under flow, adhered cells in the UBoC device were stained with Calbryte 520 AM (4 µM, #20650, AAT Bioquest) in 0.04% v/v pluronic F-127 (#P3000MP, Thermo Fischer Scientific) in clear MEM (#51200046, Thermo Fischer Scientific) solution for 45 min. After staining, the UBoC devices were immediately transferred onto a microscopic stage without flushing the staining solution. The inlet of the UBoC device was connected to a syringe pump (PHD-2000 Infusion, Harvard Apparatus) loaded with a syringe containing supplemented Hyclone MEM-α. Using fluorescence microscopy under FITC optics (IX73 inverted microscope, Olympus), a 1.69 mm^2^ field of view of stained cells in the center of the culture area was held steady. The exposure time of the camera (ORCA Flash 4.0, Hamamatsu) was manually set to 500 ms. A 2-min- long video capture depicting the cells was then started. Simultaneously, the syringe pump was also started and set to perfuse culture medium into the UBoC device at a specified flow rate. The flow rates tested were 10, 25, 50 and 100 µl min^−1^, which corresponded to approximate shear values of 0.03, 0.08, 0.17 and 0.33 dyn cm^−2^. The wall shear stress values were obtained via a CFD simulation of flow dynamics within the UBoC device (Supplementary Fig. S4) using the Creeping Flow module in COMSOL Multiphysics (ver 5.3, COMSOL, Sweden). Using Fiji ImageJ, the cells in the video were segmented and their intensity profile was tracked over time [32]. The normalized intensity profiles were then averaged, netting a flow-response curve as a function of time for the on-chip MC3T3-E1 culture.

#### Co-culture of MC3T3-E1 pre-osteoblasts and L929 fibroblasts

To perform co-culture experiments, the fluidics holder of the UBoC device was modified to allow for additional inlets and outlets for each cell line for the generation of two separate flow streams in the UBoC device (Supplementary Fig. S3), thereby enabling seeding and maintenance of multiple cell types. Otherwise, the fabrication scheme of this version was identical to the standard fluidics holder described previously (section 4.1.1).

Prior to seeding, MC3T3-E1 and L929 cell seeding solutions (both containing 7.9 × 10^5^ cells ml^−1^) were stained with red CMPTX (4 µM, #20698, Cayman Chemical) and green CMFDA dye (15 µM, #C2925, Invitrogen) solutions in clear MEM, respectively, and incubated for 30 min. After staining, both cell types were resuspended in their appropriate medium and each seeding solution was simultaneously loaded into a multi-channel pipette (45 µl, #3125000036, Eppendorf) and then seeded into the UBoC device, with integrated Ti. The seeded UBoC device was then transferred into an incubator and connected to medium reservoirs (with each inlet corresponding to the medium reservoir of the seeded cell type) and a syringe pump. The cells were allowed to adhere to the substrate for 1 h prior to the start of the flow. Subsequently, a continuous 2 µl min^−1^ medium flow was started for both medium supplies. In addition, a 10 µl min^−1^ vacuum flow via the UBoC vacuum inlet was applied for 10 min at 30-min intervals for trapped bubble removal.

On day 1 and 3 of culture, the chips were temporarily disconnected from the flow set-up and imaged under a fluorescence microscope. Directly after disconnection, the inlets and outlets of the UBoC device were blocked with stoppers to minimize the chance of infection or bubble formation. Using Fiji ImageJ, the MC3T3-E1 (red) and L929 (green) image channels were treated with a rolling ball filter (radius = 20 pixels) to improve contrast against the background [32]. The channels were merged into one image and via Fiji ImageJ, averaged intensity line profiles were extracted from the images, thereby allowing cellular distribution to be quantified [32]. Duplicates were taken for each condition and the experiment was performed twice.

### Statistics

Experimental data was presented as a mean ± standard deviation of replicate data for each sample at a given time point or category. In regards to significance testing, a two-sided one-way ANOVA analysis was performed (*α* = 0.05), with pair-wise differences and differences to control values assessed via post-hoc Tukey and Dunnett’s tests, respectively. The statistical analysis was performed using Minitab 17 software.

## Supplementary information

Supplementary information

## Data Availability

The data that support the findings of this study are available from the corresponding author upon reasonable request.
