# Supplementary material for: Universal Biomaterial-on-Chip: a versatile platform for evaluating cellular responses on diverse biomaterial substrates
Source: J Mater Sci Mater Med. 2024 Jan 11;35(1):2. doi: 10.1007/s10856-023-06771-x (PMC10784356; doi:10.1007/s10856-023-06771-x)
Supplement: Supplementary file 1 — Supplementary information [file 10856_2023_6771_MOESM1_ESM.docx]

Supporting Information

**Universal Biomaterial-on-Chip: A Versatile Platform for Evaluating Cellular Responses on Diverse Biomaterial Substrates**

*Abdul Raouf Atif, Morteza Aramesh*, Sarah-Sophia Carter, Maria Tenje, Gemma Mestres*

Division of Biomedical Engineering, Department of Materials Science and Engineering, Science for Life Laboratory, Uppsala University, 751 22 Uppsala, Sweden

E-mail: [morteza.aramesh@angstrom.uu.se](mailto:morteza.aramesh@angstrom.uu.se)

Keywords: biomaterials on chip, calcium imaging, mechanobiology, microfluidics, standardization, 3d printing

**Evaluation of L929 proliferation via LDH activity quantification**

In addition to direct manual quantification of cell numbers via counting of cells from captured fluorescence images, quantitation of LDH activity from day 5 lysed samples post-imaging was also performed. The results are displayed in Figure S4. The overall trend in LDH activity matched the cell numbers counted for day 5. Specifically, CDHA, Ti and fibrin exhibited absorbances of 1.84 ± 0.09, 1.05 ± 0.14, 1.40 ± 0.23.





**Figure S1.** Quantification of LDH activity for cell lysate samples taken on day 5 of culture. * indicates significance between associated samples (p < 0.05).

**Fabrication of cartridge for soft and porous material loading**

In the case of soft (*e.g.* hydrogel) and/or porous (*e.g.* hydroxyapatite) substrates, a carrier is needed to enable a tight gasket seal and prevent leakage. This carrier was termed as “cartridge” and is depicted in Figure S2. Similarly, as with the substrate holder, the cartridge was fabricated using FFF-3D printing in a single step process.

**
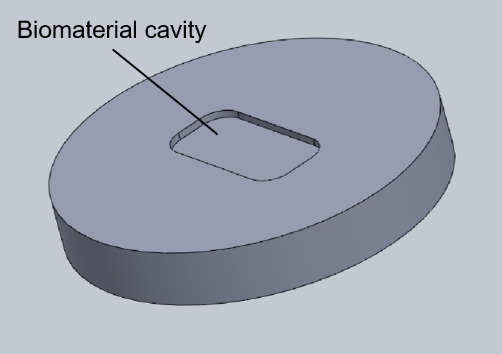
**

**Figure S2.** 3D printed cartridge for soft or porous biomaterial loading into the UBoC device.

**Fabrication of Fluidics Holder component for co-culture applications**

In order to accommodate for co-culture, the “Fluidics Holder” was modified by increasing the number of inlets and outlets to the culture area, while in-turn extending bubble removal capabilities to the added channels (Figure S3). Specifically, changes were made to the cut designs of the fluidics and vacuum PDMS layers. Otherwise, the fabrication pipeline is the same as described in the main text for the original “Fluidics Holder” in section 4.1.1.


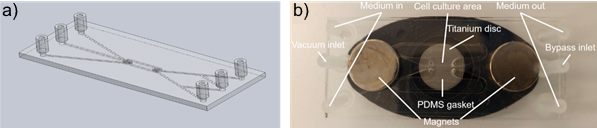


**Figure S3.** a) 3D model for “fluidics holder” modified for co-culture. b) Photograph of co-culture UBoC device.

**Simulation of flow dynamics within the UBoC device**

Using COMSOL, the geometry of the UBoC culture area was constructed (Figure S1). The Creeping Flow module was used to model the flow dynamics in the culture area at a flow of 2 µl min^-1^. The fluid used in the simulation was water (37 °C). The resultant flow pattern is shown in Figure S1. According to the color scale, the average shear stress in the center of the channel is approximately 6 x 10^-3^ dyn cm^-2^. Peak shear stress occurs at the inlet and outlet regions and is estimated to be around 11 x 10^-3^ dyn cm^-2^.


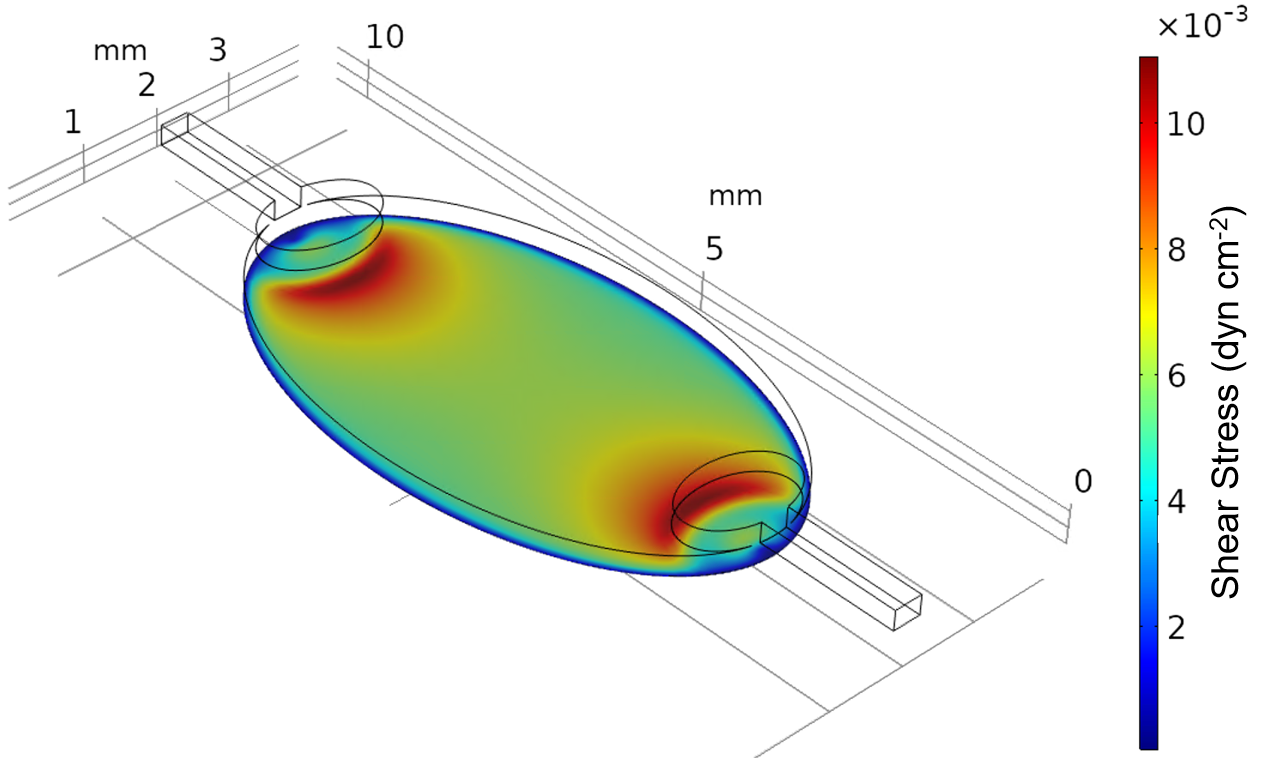


**Figure S4.** Computational fluid dynamics (CFD) simulation of a 2 µl min^-1^ flow in the cell culture channel in the UBoC device. Color gradient indicates the intensity of the shear stress in different locations within the channel.
